# Image stitching for real-time laparoscopic hyperspectral imaging

**DOI:** 10.1038/s41598-025-11687-3

**Published:** 2025-07-31

**Authors:** Marie-Sophie von Braun, Annekatrin Pfahl, Andreas Melzer, Claire Chalopin, Hannes Köhler

**Affiliations:** 1 Innovation Center Computer Assisted Surgery (ICCAS), Faculty of Medicine, Leipzig University, 04103 Leipzig, Germany; 2 Center for Scalable Data Analytics and Artificial Intelligence Dresden/Leipzig (ScaDS.AI), Leipzig University, 04103 Leipzig, Germany; 3 Institute for Medical Science and Technology (IMSaT), School of Medicine, University of Dundee, Dundee, UK; 4 Faculty of Engineering and Health, University of Applied Sciences and Arts, 37085 Göttingen, Germany

**Keywords:** Endoscopy, Biomedical engineering, Optical spectroscopy, Image processing, Imaging and sensing

## Abstract

Hyperspectral imaging (HSI) shows significant promise in the medical field for tissue detection and perfusion assessment. To extend its application to intraoperative diagnosis, laparoscopic cameras combining a high resolution color video and simultaneous HSI were developed. Spatial scanning in these cameras is performed through a push-broom motor driving a line-scan spectrograph. However, long acquisition times and the necessity of absolute immobility for patient and operator currently limit its usability in the operating room. To provide a hyperspectral acquisition alternative to the traditional push-broom motor approach, we have developed an HSI stitching pipeline that enables freehand line scanning. Our method utilizes the dual recording capability of the camera, which has both an RGB and an HSI sensor. It applies the transformations observed in the RGB video to the corresponding HSI data, then seamlessly merges this data to create a coherent panorama. This allows operators to visualize hyperspectral data as an incrementally expanding overlay on the color video by scanning the scene with the laparoscope. The pipeline evaluation confirms the generation of globally consistent and well-interpretable panoramas with a high level of detail. The registration error is not only comparable to the push-broom method but also corresponds to a real-world error of less than 0.4 mm in 95 % of the cases. Therefore, the proposed method enhances the practicability of intraoperative hyperspectral imaging by providing a dynamic, video-like experience of HSI visualizations.

## Introduction

Hyperspectral imaging (HSI) is an emerging technology in medical diagnostics, offering noninvasive and marker-free insights. By analyzing the unique spectral properties of tissues, hyperspectral imaging enables the detection of parameters like oxygen saturation and hemoglobin levels. These parameters can be visualized in false-color images and used for tissue recognition and perfusion assessment^1^. The adoption of HSI in clinical practice shows great promise for surgical guidance in robotic and minimally invasive procedures. Despite the availability of real-time multispectral imaging, HSI prevails in demanding tissue characterization due to its high spectral resolution^2^.

Initial challenges, such as bulky equipment and limited resolutions, have been addressed by the introduction of advanced systems like the $\hbox {TIVITA}^{\circledR }$ Mini Endoscopy Edition (Diaspective Vision GmbH, Am Salzhaff-Pepelow, Germany). This hyperspectral camera for minimally invasive surgery (HSI MIS) is a laparoscopic system that produces two asynchronous raw data streams: a high-resolution RGB color video and a narrow vertical “line” of hyperspectral data with high spatial and spectral resolution, covering 500–1000 nm across 100 spectral bands at 5 nm spacing. For detailed information about the HSI MIS system, please refer to the technical evaluation by Köhler et al.^2^, or to the description of the preprocessing steps and algorithms used to determine tissue parameters^3^.

Processing the two data streams captured by the HSI MIS system enables the display of both the RGB video and the hyperspectral data in side-by-side windows. However, whereas the RGB video shows the field of view (FOV) in real time, the hyperspectral display can only provide a static snapshot of the scene at a particular moment. This limitation arises from the need to scan the scene line by line for spatial information, which is achieved by an integrated stepper motor that guides the spectrograph along a linear rail. By placing these lines side by side, a static capture of the scene can be performed. Scanning the scene, however, results in long acquisition times: Depending on the resolution, the push-broom scanner requires between 4.6 s and more than 10 s to obtain a complete hyperspectral capture of the FOV. During this time, both the laparoscope and the scene must be kept absolutely still, otherwise image distortion and motion artifacts will occur. This is a major limitation for surgical applications, as organs such as the lungs or the heart perform a natural, continuous movement that cannot be halted.

Further, displaying both imaging modalities side by side does not ensure optimal surgical guidance. For precise localization of critical structures, augmenting the RGB video with hyperspectral data would be of great interest. In previous research^4^, we developed a method to visualize the physiological parameters in the spatial context of the video: By continuously registering a previously captured hyperspectral image with the current frame of the RGB video, both modalities could be overlaid in real-time. However, as soon as the camera is moved on to another part of the scene, the registration can no longer be performed and the overlay disappears. In addition, changes in the scene that have occurred in the meantime cannot be taken into account.

Facing similar constraints, Yoon et al. present a concept in^5,6^ to reconstruct a complete hyperspectral data cube from a line-scan HSI. The method uses a hyperspectral endoscopy system (HySE) that combines a CMOS camera capturing wide-field images and a single-line scanning spectrograph. After scanning the lumen with the endoscope, a panoramic image is created from the wide-field images by co-registering them in pairs. Nonetheless, although the data are acquired in real time, the hypercube is reconstructed offline. This post-processing step requires about 50 s to build a hypercube with 51 spectral channels from 59 line-scan images^5^. As a result, the technique only allows to handle very short image sequences and is not suitable for live surgical guidance.

To address the shortcomings of both the HSI MIS and the HySE system, we developed an HSI processing algorithm that offers an alternative to the push-broom capture method. By manually sliding the laparoscope laterally across the region of interest, the user generates a series of HSI lines. Our algorithm combines (“stitches”) all lines successively and builds a dynamically expanding panorama of hyperspectral data as the laparoscope moves. To visualize both modalities together, the panorama is overlaid onto the RGB video recorded by the RGB camera. We assessed the performance of the implemented method by evaluating its subjective quality, accuracy, and the robustness of hyperspectral reconstruction.

## Stitching pipeline

Image stitching describes the process of combining overlapping images with differing alignments, orientations, or illuminations into a single panorama. The rationale behind image stitching involves establishing the relative orientation of images, aligning them based on this orientation, and finally assembling them on a common canvas^7^.

In our case, direct stitching of HSI lines was not feasible, as the extreme narrowness of the HSI lines makes it impossible to determine their alignment to each other. Instead, our method leverages the simultaneous recording of the FOV by both RGB and HSI sensors. This shared perspective enables the transfer of laparoscope movements captured in the RGB video directly to the corresponding HSI lines. Therefore, the process unfolds as follows. First, we quantify the transformation between two RGB frames. This step is referred to as *registration* and involves identifying changes in orientation (rotation, translation, scaling, and shearing), in order to establish geometric correspondence between the frames. Next, we apply this transformation to the HSI lines that were recorded in the same period. Finally, we merge the transformed HSI lines into a panorama, ensuring a coherent visual representation.

Given the real-time requirements, only incremental stitching techniques, which adapt and integrate data as they are captured, can be employed. Several concepts for incremental global registration^8,9^, or for combined local and global registration^10,11^ have been introduced in the past. The proposed algorithm employs the hybrid method from^12^, dividing the registration into an initial rough frame-to-frame approximation and a refined frame-to-panorama registration using a panorama keypoints database.

To leverage the parallel processing capabilities of a multi-threading approach, we segmented the pipeline into independent modules, with each module responsible for a different processing phase on its own thread. The modules are discussed individually in the following sections. It should be noted that all default parameters described therein were determined empirically on cardboard test targets during development. These values need to be individually configured to adapt the pipeline to the varying requirements of different use cases or changes in hardware.

The pipeline was developed using Python 3.8, with multi-threading and GUI managed by Qt^®^^13^ version 5.15. The OpenCV library^14^ version 4.5 was used for most of the image processing tasks.

### Preliminary steps

First, the entrance slit of the spectrograph was fixed in position to capture a narrow, one-pixel-wide vertical line of hyperspectral data in the center of the FOV. Further, several measures were necessary to accommodate the cameras’ characteristics. We corrected the laparoscope’s barrel distortion by generating a calibration map, which was precomputed once during setup and subsequently applied in real time to undistord each incoming RGB frame. To address the slight angle discrepancies between the RGB and HSI sensors, we established a homography to align their image planes. This process yielded a sensor homography matrix, denoted as $\varvec{H}_{sens}$. Additionally, since the position of the entrance slit of the spectrograph was fixed, we accounted for the horizontal shift of the line from the left edge to the center of the RGB image, which was represented as the homography $\varvec{H}_{pos}$.

**Fig. 1:**
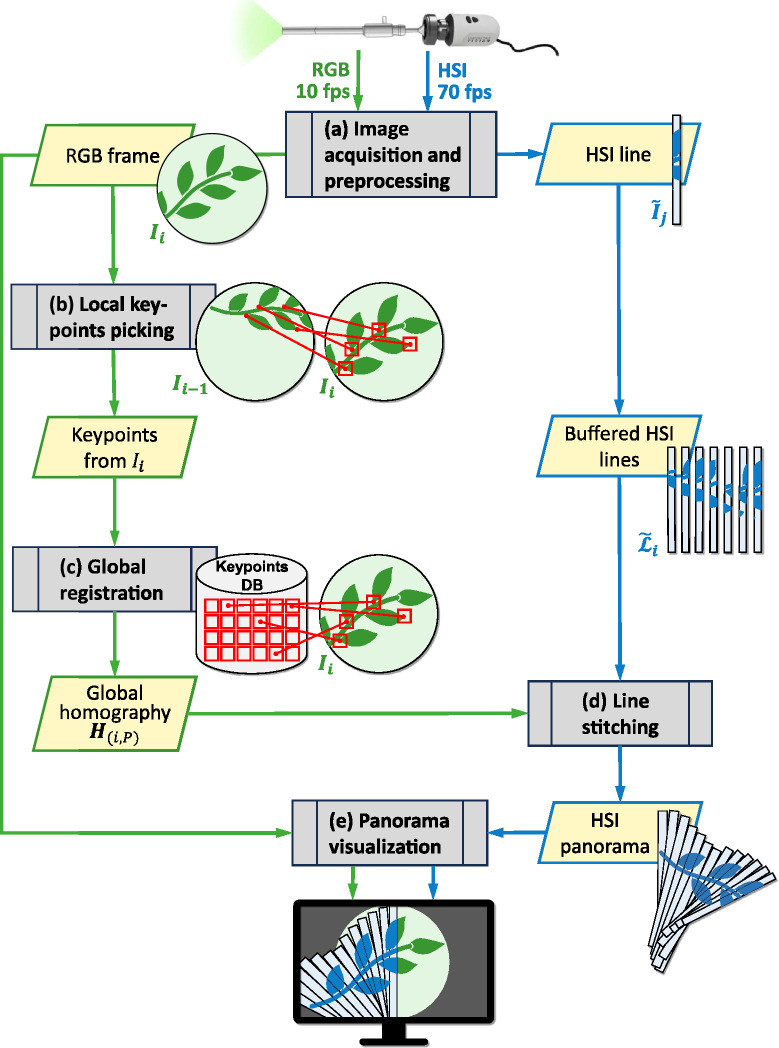
Flowchart of the stitching pipeline. Modules are depicted in gray, with data transfers indicated by yellow parallelograms. RGB frames are represented in green, and HSI in blue. Note that HSI lines are depicted with an illustrative width for clarity, although they are 1 pixel wide in reality. Timestamps are omitted for simplification. **(a)** This module receives and preprocesses two asynchronous raw data streams from the laparoscope and forwards them separately. **(b)** The picking module extracts salient keypoints from the current RGB frame $I_i$ and matches them with its predecessor $I_{i-1}$. Sanity checks associated with this frame-to-frame registration enable the selection of the best keypoints. **(c)** The coordinates and descriptors of the most robust keypoints from frame $I_i$ are transferred to the global registration module, which maintains a database of accumulated keypoints since sequence start. A brute force matcher establishes correspondence between current and stored keypoints, enabling computation of a global frame-to-panorama homography ${H}_{(i, P)}$. **(d)** The homography is adjusted for application on HSI lines and used to warp and stitch the buffered set of corresponding lines $\tilde{\mathcal {L}}_{i}$ onto the panorama. **(e)** The visualization module renders the HSI panorama as an overlay on the RGB video. *This figure has been designed using an icon from Flaticon.com*

### Image acquisition and preprocessing

The *image acquisition and preprocessing module* simultaneously handles the two asynchronous raw data streams sent by the laparoscope to a Windows 10 computer via a CAT 5 Ethernet cable. Both cameras in the HSI MIS system can record at a maximum rate of 120 fps. However, due to bandwidth limitations of the Ethernet connection, the RGB image video had to be reduced to 10 fps, and the hyperspectral stream to 70 fps. These values were empirically determined as being robust against fluctuations and showing satisfying results without overloading the connection.

In addition to the preprocessing steps described in^3^ and^2^, the calibration map is applied to the RGB images to undistort them. Further, a contrast-limited adaptive histogram equalization (CLAHE, clip limit: 2.0, tile grid: 8 $\times$ 8)^15^ is conducted to mitigate varying illuminations and intensity inhomogeneities. To ensure later synchronization between the images of both streams, each RGB image and each hyperspectral line is coupled with a timestamp documenting the moment of its arrival from the laparoscope system. Therefore, the image acquisition and preprocessing module yields two distinct asynchronous output streams (Fig. 1a):

- Pairs consisting of an undistorted, contrast enhanced RGB image of dimensions 960 $\times$ 540 pixels (width $\times$ height) and its respective timestamp. The former is hereafter referred to as *RGB frame*, with the $i{\rm th}$ frame denoted by $I_i$. These pairs are passed to the *picking* module.
- Pairs consisting of a line of hyperspectral data, hereafter referred to as *HSI line* and denoted by $\tilde{I}_j$ (for the $j{\rm th}$ line since sequence start) of dimensions 1 $\times$ 540 $\times$ 100 pixels (width $\times$ height $\times$ spectral) and its respective timestamp. These pairs are passed to the *stitching* module.

In subsequent sections, a parameter *p* is denoted by its simple notation when it is related to RGB data, and by $\tilde{p}$ when related to HSI data.

### Local keypoint picking

To register two frames effectively, one approach involves detecting salient keypoints in the images and describing each of them, in order to match the corresponding features. This is the role of the *picking module*, which extracts and selects (“picks”) keypoints from the incoming RGB frames (Fig. 1b).

Each new incoming frame undergoes initial processing using an Oriented FAST and Rotated BRIEF (ORB) detector^16^, extracting the 1000 most salient keypoints. All keypoints are saved with their corresponding pixel coordinates (*x*, *y*), referenced to a coordinate system with origin in the upper left-hand corner of the RGB frame. ORB computes a binary descriptor for each keypoint, resulting in two lists per frame: one containing keypoint coordinates and the other their respective descriptors. The module keeps track of the keypoints’ coordinates and descriptors for two frames: the latest captured RGB frame $I_i$ and its predecessor $I_{i-1}$.

Correspondences between the keypoints are then established and a brute force matcher using Hamming norm as a distance measurement produces a list of matches between both sets. This list is subsequently refined using a Lowe’s ratio of 0.8.

The final stage of the local registration process consists of estimating the transformation that maps the matched keypoints. For this purpose, it is assumed that the laparoscope camera has the geometric properties of a pinhole camera model and that the movements of the laparoscope are small enough to approximate the observed scene by a plane. Thus, the mapping can be approximated by a local transform homography^17^, mapping the common keypoints of the current frame to their counterparts in the preceding frame:

1 $$\begin{aligned} \varvec{H}_{(i, i-1)}: I_{i} \mapsto I_{i-1}. \end{aligned}$$

The pixel coordinate origin of the previous frame serves as the reference for transformation, with the current frame being the image to warp. The homography is estimated using a RANdom SAmple Consensus (RANSAC) algorithm^18^ with a confidence level of 0.995 and a threshold of 8.0 pixels.

Locally registering two consecutive frames may seem superfluous, as this first homography will be replaced by a more accurate global homography in the next module. Nevertheless, this serves three key objectives:

- *Improving the quality of the keypoints*. Using RANSAC early for outlier detection identifies higher-quality keypoints, ensuring only robust matches are utilized for global alignment in the registration module.
- *Identifying motion*. The homography estimate identifies image motion. Running the stitching pipeline with minimal camera movement wastes computing resources and enlarges the keypoints database unnecessarily. Assessing spatial displacement between frames helps decide if there’s sufficient change for further processing.
- *Filtering low-quality frames*. The local homography estimation in the pipeline helps filter out low-quality frames through plausibility checks, preventing problems caused by insufficient overlap or textureless regions. This process involves ensuring a sufficient number of 40 inlier matches for robust detection, evaluating image motion for excessive displacement, and checking for abnormal frame distortion due to the transformation. Frames failing the quality tests are discarded. After 20 invalid attempts, the panorama is reset and a new stitching sequence begins. Otherwise, the picking module forwards a tuple containing the keypoint coordinates, descriptors, and timestamp of the RGB frame to the registration module.

### Global registration

The *registration module* establishes global registration between the last RGB image and the current panorama. Global registration is favored over local registration as the latter can lead to drift from error accumulation. However, continuously saving the entire RGB panorama and extracting its keypoints to register with a new frame would be computationally prohibitive. Instead, the module maintains a database containing coordinates and descriptors of its strongest keypoints since the start of the sequence (Fig. 1c).

#### Global homography

In a first step, a brute force matcher, similar to the one operating in the picking module, matches the previously extracted keypoints of the current frame with the keypoints stored in the database. The coordinates of the keypoints are used to compute the global homography mapping the keypoints of the current frame to their counterpart in the panorama:

2 $$\begin{aligned} \varvec{H}_{(i, P)}: I_{i} \mapsto P, \end{aligned}$$

where *P* denotes the panorama described by the keypoints database in its most recent state. A RANSAC algorithm identifies inlier matches and $\varvec{H}_{(i, P)}$ undergoes plausibility tests similar to local registration. If deemed plausible, the homography is sent to the stitching module for HSI line alignment based on the estimated global transformation.

#### Database maintenance

After each processed RGB frame, the database undergoes a maintenance step. For each keypoint in a new frame, the matching process for global registration results in three possible outcomes:

- *No match*: the keypoint is considered new and *added* to the database after aligning with the general coordinate system using the homography $\varvec{H}_{(i, P)}$. This system is set by the upper left corner of the first incoming RGB frame at the beginning of the stitching sequence and does not change thereafter.
- *Inlier match*, contributing to a plausible homography, likely describes the same physical point in the scene. Assuming that a more recent description of a keypoint is more reliable than an older one, the coordinates and the descriptor are *updated* in the database.
- There is a match, but it is either deemed to be an *outlier match* after the RANSAC algorithm, or it is involved in the computation of an *implausible homography* matrix: The keypoint is considered a potential uncertainty factor and is *discarded*.

The database is regulated to prevent excessive growth. Key measures include selecting only the 500 most relevant matches based on Hamming distance for global homography calculation, adding only the 50 strongest unmatched keypoints to the database, retaining the 200 inlier matches with the lowest Hamming distance, and removing keypoints that did not match after 80 iterations. These steps ensure consistent computational speed, essential for meeting real-time requirements.

### Line stitching

The *stitching module* aims to apply the previously computed homography transform to the temporally corresponding HSI lines in order to position them accurately on the panorama canvas (Fig. 1d). It inputs two asynchronous data streams:

- Pairs of the global homography, mapping the current RGB frame to the panorama, and its timestamp. The pair is denoted by $(\varvec{H}_{(i, P)}, t_{i})$ and arrives from the registration module at 10 fps by default.
- Pairs consisting of an HSI line and its timestamp. The pair is designated $(\tilde{I}_{j}, \tilde{t}_{j})$ and arrives from the image acquisition module at 70 fps. These tuples are buffered until the tuple homography–timestamp arrives, which triggers the stitching mechanism.

#### Selection of the HSI lines

First, we have to determine which lines coincide in time with the current RGB frame. At the default frame rates of 70 fps (HSI) and 10 fps (RGB), about 7 HSI lines are captured between two consecutive RGB images. The timestamps help pinpoint these lines within the buffered list as the set $\tilde{\mathcal {L}}_{i}$ of lines captured between the frames $I_{i-1}$ and $I_{i}$:

3 $$\begin{aligned} \tilde{\mathcal {L}}_{i}=\{\tilde{I}_{j}\ |\ t_{i-1} \le \tilde{t}_{j} + \delta < t_{i}\}, \end{aligned}$$

$\delta$ being the correction of a slight time delay between the raw data of both streams that was determined empirically as being approximately 35 ms.

#### Preparation of the HSI lines

The aim of the HSI MIS pipeline is to visualize physiological tissue parameters through hyperspectral imaging. However, as most captured wavelengths are invisible to the human eye, the data must be transformed into false-color images. Synthetic colors, derived from measured reflectance and tailored to the desired vital parameter, create a false-color line of size 1 $\times$ 540 in the spatial dimension.

#### Homography adjustment

The homography $\varvec{H}_{(i-1, P)}$ corresponds to the first line in $\tilde{\mathcal {L}}_{i}$. For the subsequent lines $\tilde{I}_{k}$ in $\tilde{\mathcal {L}}_{i}$, linear interpolation to the next homography is applied:

4 $$\begin{aligned} \varvec{H}_{(k, P)} = \biggl (1-\dfrac{k}{|\tilde{\mathcal {L}}_{i}|}\biggr ) \varvec{H}_{(i-1, P)} + \dfrac{k}{|\tilde{\mathcal {L}}_{i}|} \varvec{H}_{(i, P)}, \end{aligned}$$

with $k \in \{0, 1,..., |\tilde{\mathcal {L}}_{i}|-1\}$. $\varvec{H}_{(i-1, P)}$ is initialized as the identity matrix at the beginning of the stitching sequence.

These homographies, though, describe transformations that are valid for $960 \times 540$ pixels RGB images and cannot be directly applied to the centered HSI line due to differing coordinate origins and sensor displacement. This was considered in the preliminary steps by determining the adjustments $\varvec{H}_{pos}$ and $\varvec{H}_{sens}$. Therefore, the coordinate system must be adjusted before $\varvec{H}_{(k, P)}$ can be applied to a HSI line. This is done by matrix concatenation:

5 $$\begin{aligned} \varvec{{H'}}_{(k, P)} = \varvec{H}^{-1}_{pos} \varvec{H}^{-1}_{sens} \varvec{H}_{(k, P)} \varvec{H}_{sens} \varvec{H}_{pos}. \end{aligned}$$

This equates sequentially converting the coordinate system from a centered HSI line to a RGB frame, applying the homography mapping, and reverting the coordinate system back to that of the centered HSI line.

#### Offset calculation

The panorama’s canvas is then expanded to fit the HSI lines. When initiating a stitching sequence, the panorama’s coordinate system is set by the first line, aligning the origin with its upper left corner. This origin remains as long as no new line has to be placed left or above this first line. In this case, the coordinates of the line would hold negative values, which is not compatible with image coordinates. If needed, the origin of the canvas is therefore shifted by an offset in *x* and/or *y* direction. This translation can again be expressed as a homography, denoted for the line $\tilde{I}_{j}$ by $\varvec{H}^T_{j}$, which is accumulated over time by means of matrix multiplication. This allows the panorama to grow on all sides and dynamically update the coordinate origin as its upper left corner.

#### Adaptive line width

The HSI line has a native width of one pixel. Stitching such narrow lines at 70 fps, requires slow scanning speed to ensure complete scene coverage. To bridge the gaps between the lines, the HSI line’s width can be artificially expanded through the duplication of its intensity values $\tilde{w}$ times along the horizontal spatial dimension. However, selecting an appropriate value for $\tilde{w}$ is a challenging task. Line duplication corresponds to piece-wise constant interpolation, introducing inherent inaccuracies. Consequently, the line width should be kept as minimal as possible while meeting the scanning speed’s requirements. To find the best compromise, we developed a dynamic approach that increases the line width in sync with the scanning speed. The rationale for this approach is to ensure that the width of a line is always at least as large as the distance to its successor.

This distance is determined by the amount of movement since the last frame, which is given by the transformation $\varvec{H}_{(i, i-1)}$. Its equivalent for HSI lines can be determined by using the global homography of the current and its inverse for the past iteration:

6 $$\begin{aligned} \varvec{H'}_{(i, i-1)} = \varvec{H'}^{-1}_{(i-1, P)} \varvec{H'}_{(i, P)}. \end{aligned}$$

The horizontal translation induced by $\varvec{H'}_{(i, i-1)}$ is calculated for the four corner points of an HSI line. Let $(x_m, y_m)$ and $(x'_m, y'_m)$ be the coordinates of such a corner $c_m$ before and after transform, respectively, with $m \in \{0, 1, 2, 3\}$. Then, the translation on the *x*-axis of this corner is computed as the absolute value $\big | x'_m - x_m \big |$. The largest translation among the four corners is denoted as $T_i$, which represents the maximal horizontal gap in pixels that has to be covered by the elements of the set $\tilde{\mathcal {L}}{i}$. It is then divided by the number of elements in the set and rounded up to the nearest whole number:

7 $$\begin{aligned} \tilde{w}_{i} = \Big \lceil \dfrac{T_{i}}{|\tilde{\mathcal {L}}_{i}|}\Big \rceil . \end{aligned}$$

$\tilde{w}_{i}$ is thus the ideal width for the lines contained in $\tilde{\mathcal {L}}_{i}$. In practice, the width should not exceed a maximum value, so that it is capped at a default threshold of 8 pixels. All lines in the buffer are duplicated horizontally, now having a spatial dimension of $\tilde{w}_{i}$ $\times$ 540 (width $\times$ height).

#### Line warping and stitching

Eventually, each line contained in $\tilde{\mathcal {L}}_{i}$ is warped according to its associated homography. This homography maps each pixel of the line to its position in the new panorama. Let $\tilde{I}_{k}$ be the $k{\rm th}$ line in $\tilde{\mathcal {L}}_{i}$ and the $j^{th}$ line in the stitching sequence. Then, by combining the term given by (5) with the offset homography $\varvec{H}^T_{j}$, the final transform used to warp $\tilde{I}_{k}$ is computed as

8 $$\begin{aligned} \varvec{H}^{final}_{k} = \varvec{H}^T_{j} \varvec{H'}_{(k, P)} = \varvec{H}^T_{j} \varvec{H}^{-1}_{pos} \varvec{H}^{-1}_{sens} \varvec{H}_{(k, P)} \varvec{H}_{sens} \varvec{H}_{pos}. \end{aligned}$$

The warped lines in $\tilde{\mathcal {L}}_{i}$ are then aligned onto the canvas previously expanded to the correct dimensions if necessary. If areas overlap, the newer line replaces the older one in order to ensure that the panorama is always up to date. In this manner, with each new RGB image, the corresponding HSI lines are added to the canvas, thus making the panorama grow incrementally in real-time.

To ensure that the panorama does not grow uncontrollably over time, leading to increasing memory and computation time costs, a “forget-function” was implemented. After each new RGB frame, the area of the panorama to be displayed on the GUI is estimated. If needed, any sections of the panorama extending more than 100 pixels beyond the display window are cropped.

To finish the iteration, the stitching module sends a pair containing the panorama and the final homography used for stitching the most recent line to the visualization module.

### Panorama visualization

The role of the *visualization module* is to render the panorama as an augmentation of the RGB video (Fig. 1e). At this stage of the pipeline, the panorama has the orientation of the first line in the stitching sequence, despite possible movements during stitching. Therefore, to overlay the panorama over the current RGB image, it must first be adjusted to the current viewing angle of the camera. This step is achieved by transforming the panorama with the inverse of the homography sent by the stitching module, before cropping it to the dimensions of the monitor view. Both images are then superimposed on each other by means of a linear blend operator. The overlay represents the augmentation of the current RGB frame with reconstructed hyperspectral data. Displaying such overlays at 10 fps allows the user to visualize the growing panorama of hyperspectral data in a video-like experience.

Depending on preference or application, the user can switch between display modes in the system: RGB video with full temporal and spatial resolution, static HSI push-broom capture, or dynamic freehand HSI using the stitching pipeline.

## Evaluation methods

### Experimental setup

**Fig. 2:**
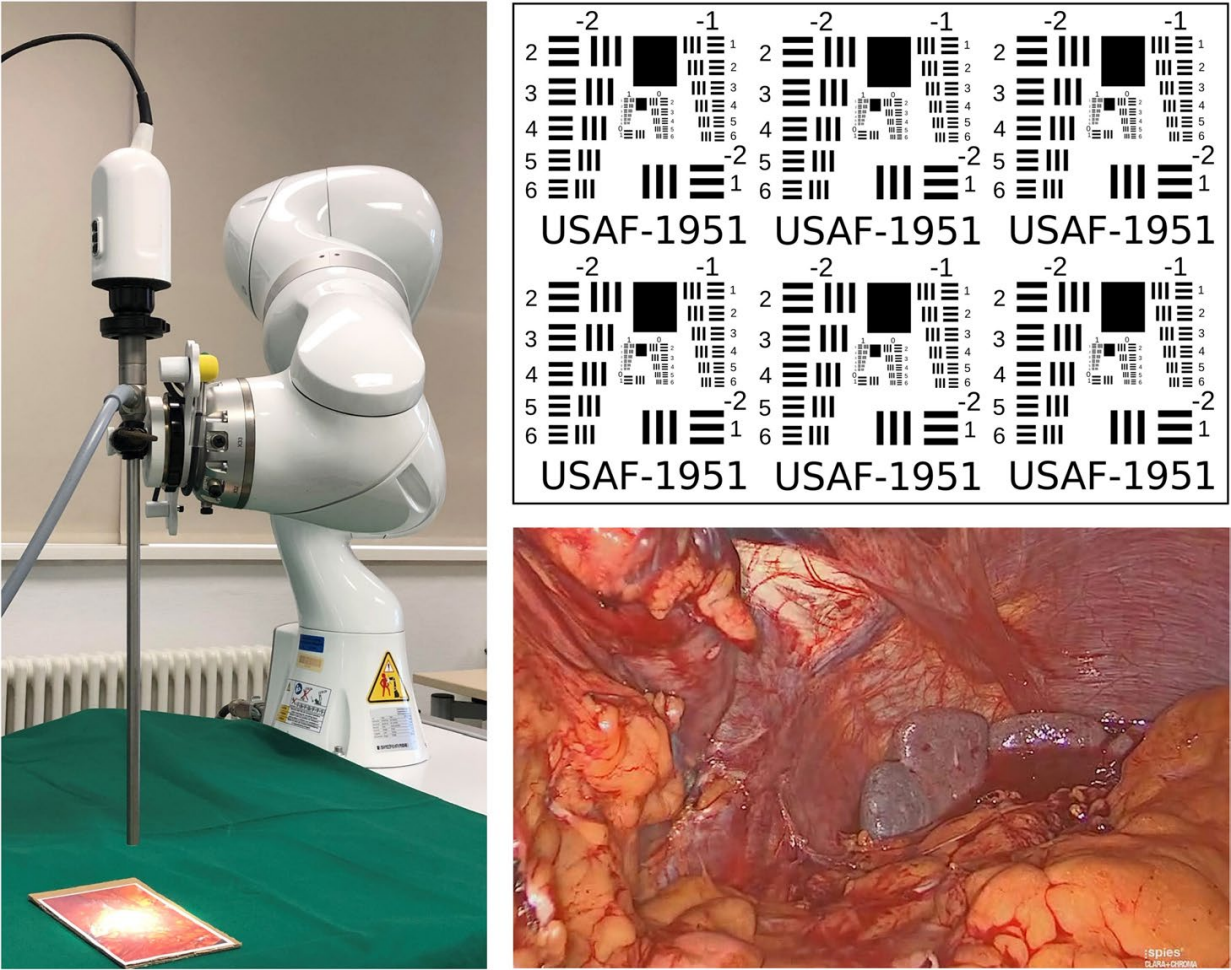
Experimental setting for the robot-guided evaluation scans. Left: laparoscope mounted to a KUKA arm. Top right: USAF 1951 target. Bottom right: image frame from laparoscopic visceral surgery video.

Recording tests were performed using the 2D targets displayed in Fig. 2: a USAF 1951 test target^19^ and a frame extracted from a laparoscopic visceral surgery video^20^. Both targets were printed on cardboard in two sizes to accommodate varying working distances: the USAF target at 65 $\times$ 69 mm and 85 $\times$ 90 mm, each duplicated to fill a DIN A4 page, and the surgery image at 140 $\times$ 80 mm and 210 $\times$ 148 mm.

#### Robotic scans

We first carried out a series of tests to investigate the pipeline’s suitability for use in robotic surgery. For this, the laparoscope was mounted to a KUKA LBR iiwa 7 R800 robotic arm^21^ (Fig. 2). The robot arm replicated scanning paths with the camera at a velocity of 10 mm/s. Different paths were programmed to assess the algorithm’s robustness against various geometric transformations. To determine whether the distance between target and laparoscope affects the quality of the reconstructed HSI panorama, the test series were performed twice: at a working distance of 35 mm and at 60 mm, leading to a total of 28 image sequences of about 20–30 seconds.

To explore the impact of the scanning speed on the quality of the reconstruction, experiments with the USAF target were performed at different speeds. The robot arm’s velocity was programmed at increments from 5 mm/s to 100 mm/s. For these tests, the camera followed a 70 mm horizontal path, recording successively at working distances of 35 mm and 60 mm.

#### Push-broom scans

For a direct comparison between reconstructed robotic arm panoramas and push-broom images, ten static captures of the USAF target were made using both RGB imaging and static HSI hypercubes, with the latter acquired via the integrated push-broom motor. The scanning speed of the push-broom motor was estimated by timing the capture duration and measuring the target area covered.

#### Freehand scans

The second series of tests focused on freehand scans to mimic real-world scenarios. The cardboard targets were positioned on a table, and recordings were made while standing, using a hand-guided motion that combined all geometric transformations. The working distance varied between approximately 30 mm and 60 mm. Unlike the robotic series, the scanning speed was variable and estimated to fluctuate between 5–20 mm/s. Four freehand scans of about 30 seconds each were reconstructed.

Throughout all scanning processes, except for the push-broom image captures, the laparoscope simultaneously recorded an RGB video at 10 fps and a raw hypercube consisting of all hyperspectral lines at 70 fps. Despite the feasibility of real-time execution, these files were saved to a hard disk, and the stitching operations for the test series were conducted offline to facilitate measurements and retrospective analyses. In the experiments, the adaptive line width feature was disabled to ensure consistent spatial alignment with the acquired data, enabling reliable manual keypoint annotation during evaluation. The fixed width of 3 pixels was selected as a compromise between visual interpretability and annotation accuracy.

#### Computational speed

To assess real-time capability, we measured the processing time per RGB frame for each core module across 20 additional sequences of 65 s each (total of approx. 12,500 RGB frames), conducted under the same conditions as the freehand scans. These times were converted to fps to quantify module-wise throughput. This breakdown reflects the pipeline’s multi-threaded design, which allows parallel execution of independent processes, and helps identify potential bottlenecks. The computational time of the threads for image acquisition and preprocessing was not measured, since their real-time capacity was already known from previous studies^2^. In addition, we analyzed how processing times evolved over time, in order to assess whether computational or memory demands increased during extended operation.

### Qualitative examination

To evaluate the perceived quality of the stitching algorithm, the resulting HSI panoramas of the test series were presented. Since displaying the physiological parameters is not only irrelevant for evaluation, but even hinders visual perception, synthetic gray colors were used to mimic a grayscale RGB image. The first impression in terms of global realism and perception of details was reviewed. Further, potential artifacts, distortions, or other representation errors were sought.

### Accuracy metrics

Given our objective of augmenting RGB video with HSI data through a semitransparent overlay, our evaluation centered on the panorama’s geometric consistency, and particularly its alignment with the corresponding RGB frame, serving as the ground truth. For this purpose, keypoint pairs representing identical physical landmarks were manually marked on both RGB images and their corresponding HSI panoramas.

Registration accuracy was calculated using the Euclidean distance between each pair of keypoints. The median of these distances across all pairs and images provided the overall registration error, reported in pixels (px) with interquartile range (IQR, Q1–Q3). This calculation assessed sequences across varying camera paths, hyperspectral image acquisition methods, and scanning speeds, analyzing error distribution across these factors. To determine the clinical relevance of the computed error, we further converted the registration error from pixels to millimeters by cross-multiplying the width in pixels of the HSI image and the width in millimeters of the corresponding target section.

## Results

### Qualitative examination

**Fig. 3:**
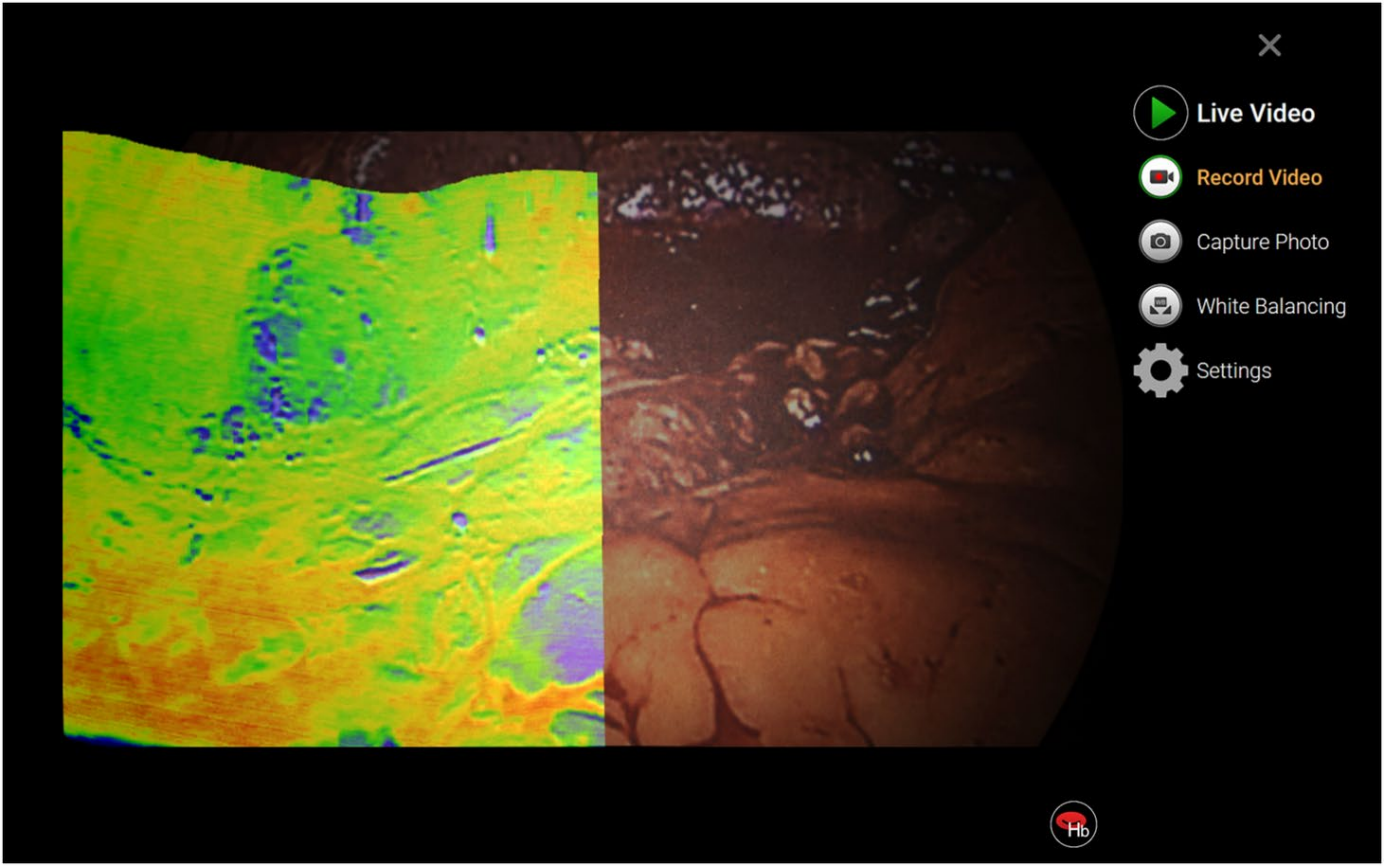
Screenshot of the GUI taken during freehand scanning, showing a reconstructed false-color HSI panorama augmenting the RGB video.

**Fig. 4:**
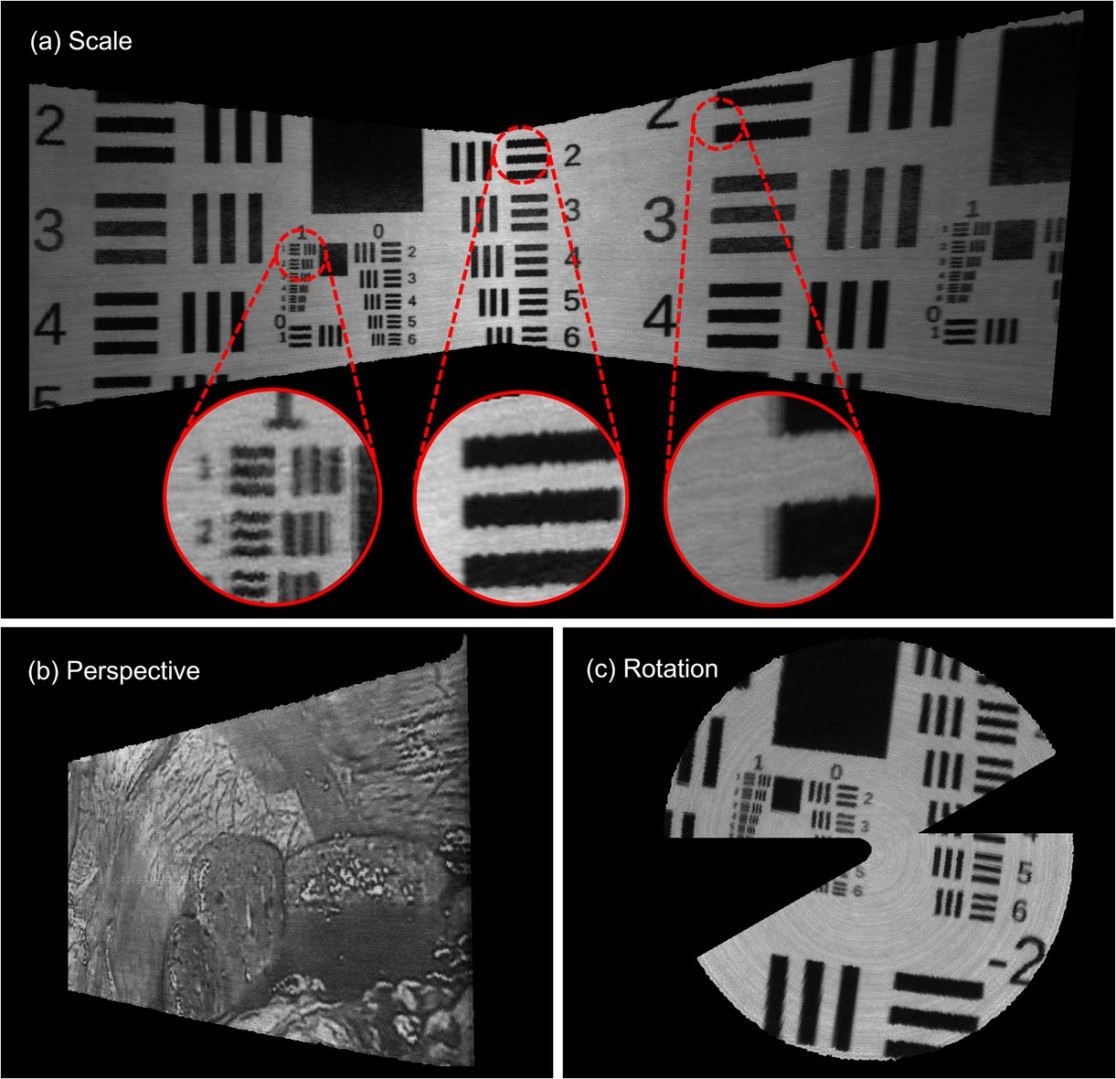
Examples of HSI panoramas reconstructed from robot arm scans, presented in synthetic grayscale, and categorized based on the robot arm’s camera path. The red circles show cut-outs for a detailed visualization of artifacts. Working distance: 35 mm (**a,b**) and 60 mm (**c**).

Figure 3 presents a screen capture taken during a freehand scan. Videos illustrating the incremental generation of such overlays can be found as supporting material to this publication. Furthermore, Fig. 4 shows examples of panoramas obtained using a robotic arm, illustrating precise target reproduction despite geometric transformations induced by the camera path. The patterns are clear, undistorted, and correctly proportioned. However, upon closer inspection, minor artifacts emerge: “jagged” elements, where straight lines appear zigzag (left and middle circles in Fig. 4), and a ghosting effect, where elements show a slightly offset shadow (left and right circles). These artifacts are more pronounced in the high-contrast USAF target images.

**Fig. 5:**
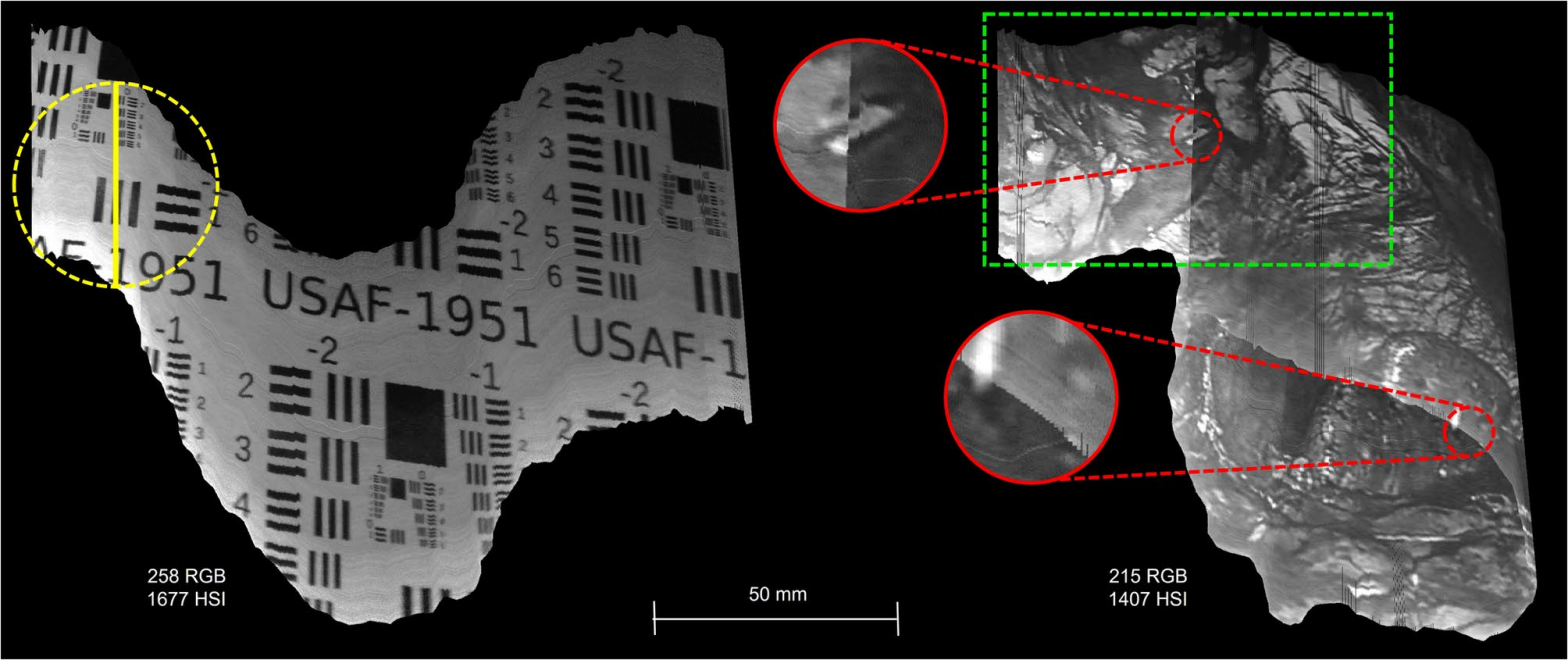
Reconstructed HSI panoramas, obtained from freehand scans and displayed in synthetic gray scales. The figures indicate the number of RGB frames and HSI lines processed in each sequence. A scale at the bottom gives an indication of the size of the scanned area on the physical target. Left: The laparoscope scanned along a sinuous path from left to right with progressive zoom-out. The yellow dashed circle represents the laparoscope’s circular field of view; the solid yellow line within it marks the position of the HSI acquisition line at that moment. Reconstructed target section: approximately $15\times 12$ cm. Right: Path starting at the top left, turning around at the bottom right and ending at the top center. Red circles show cut-outs for a detailed visualization of artifacts. The left side of the green rectangle corresponds to an area with overly high registration errors. Reconstructed target section: approximately $11\times 13$ cm.

Figure 5 shows two panoramas from freehand scans, with observations similar to those from robot-recorded panoramas. Additionally, two new artifact types are noted: noticeable intensity differences at overlaps (red circles in Fig. 5) and slight displacements in sections scanned at different times, causing offsets in continuous elements (top circle in Fig. 5).

During scanning speed assessments at 35 mm working distance, the algorithm could not reconstruct meaningful panoramas at speeds higher than 50 mm/s. Thus, these sequences were excluded from further quantitative evaluation. The problem was not observed in sequences recorded at a greater distance.

**Fig. 6:**
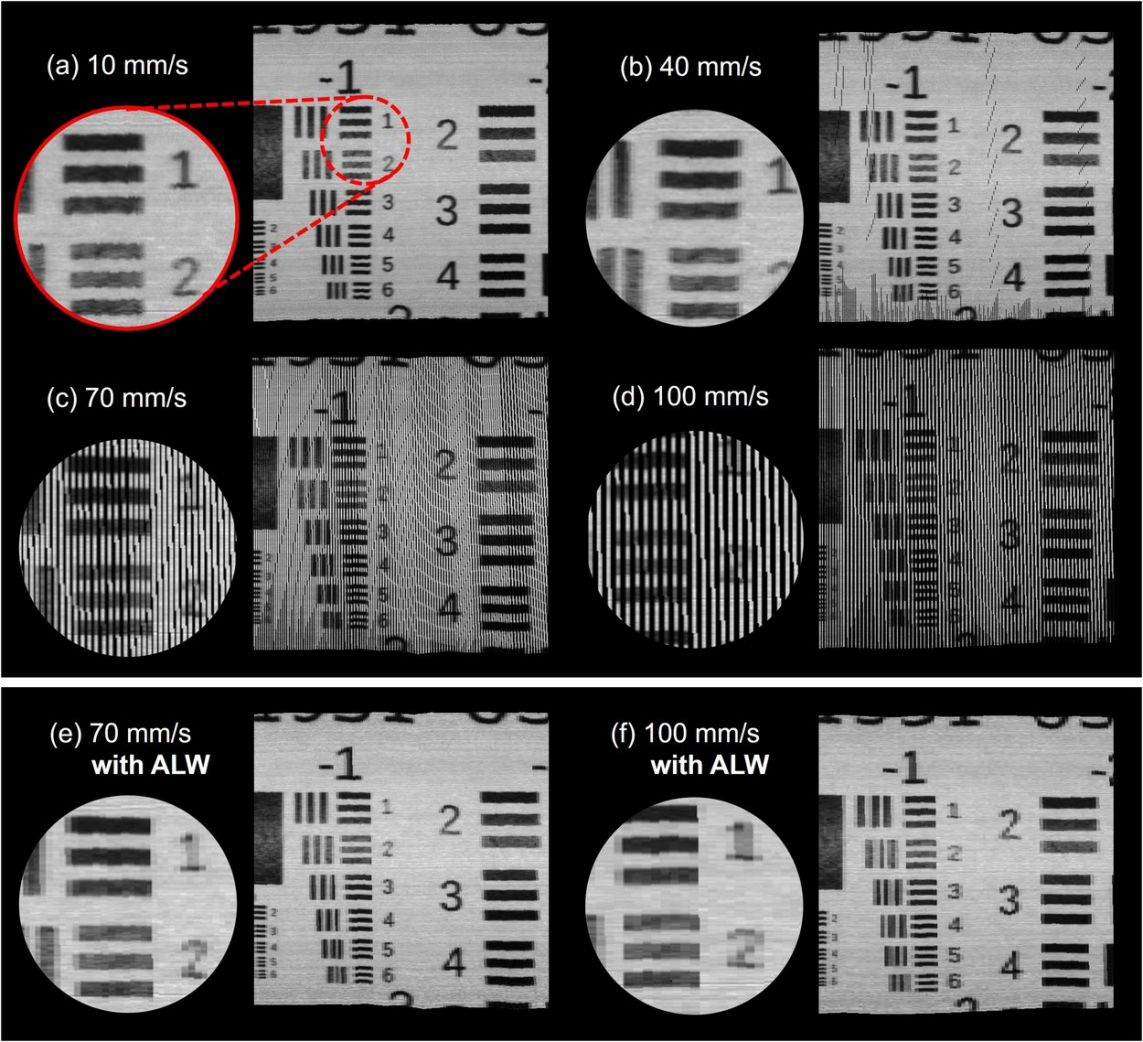
HSI panoramas, reconstructed at various scanning speeds. With increasing speed, the HSI lines are too far apart to be stitched together seamlessly, resulting in black gaps between the lines (**b–d**). However, panoramas (**e,f**) show that this problem can successfully be solved by the adaptive line width (ALW).

At a 60 mm working distance, as shown in Fig. 6 (a–d), quality visibly declines with increased speed, evidenced by emerging black regions between lines and a slight “jagged” appearance in straight lines. Overall, scans from 35 mm distances were more negatively impacted than those at 60 mm. The adaptive line width, designed to eliminate these black gaps, is shown in Fig. 6 (e, f) to effectively resolve the problem. Additionally, slower scans with enabled adaptive line width resulted in reconstructions with line widths typically between 1 and 2 pixels, potentially enhancing registration accuracy over the fixed 3-pixel width of the non-adaptive approach, though this was not quantitatively assessed.

### Registration error

Quantifying the hyperspectral reconstruction quality is a major element in the evaluation of the stitching pipeline. For this purpose, the influence of various factors on the registration error was investigated.

#### Impact of the geometric transformation

**Fig. 7:**
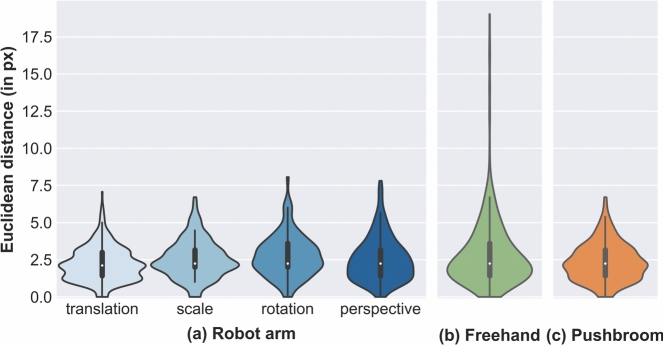
Distribution of the registration error with regard to the different acquisition methods, combining both working distances for (**a,c**). The robotic scans are organized by camera path; freehand scans integrate all transformations; push-broom scans involve only translations. The error is defined by means of the Euclidean distance in pixel between manually annotated, corresponding keypoints on RGB frames and HSI panoramas. The panoramas of (**a,b**) were reconstructed via image stitching. Samples sizes: robot arm 1200, freehand 400, push-broom 200 keypoint pairs.

Figure 7a displays for each camera path the distribution of the registration errors between the RGB frames and the corresponding HSI panoramas. The key statistics show that the errors are similarly distributed across the different paths, with medians ranging from 2.1 px (IQR: 1.4–3.0) to 2.2 px (IQR: 2.0–3.6). Due to a few outliers, the maxima are slightly higher for rotations (8.1 px) and perspectives (7.8 px) than for translations (7.1 px) and scales (6.7 px).

#### Impact of the acquisition method

The accuracy of robot arm scans, freehand scans, and integrated push-broom captures is presented in Fig. 7, combining all working distances where applicable. After aggregating the camera paths, robotic and push-broom scans show similar error distributions with identical quartiles Q1, Q2, Q3 at 1.4 px, 2.2 px, and 3.2 px, respectively. Differences arise in outliers and maxima (8.1 px for robotic scans vs. 6.7 px for push-broom).

For the freehand scans (Fig. 7b), Q1 and Q2 align with those of the other methods, while Q3 is slightly higher at 3.6 px. In contrast, the maximum error for freehand scans peaks notably at 19.0 px, with 13 outliers among 400 keypoint pairs–eleven of which exceed the combined maximum error of the robotic and push-broom methods. Notably, all these outliers are concentrated in the same section of a single panorama.

To estimate the real-world correspondence of these errors, we assumed an average working distance of 50 mm. At this distance, the 960 px wide RGB image shows a section of the target of 85 mm width. Therefore, the identical medians of the three acquisition methods (2.2 px) correspond to a distance of 0.2 mm in the real-world, and the maximum error of 19.0 px translates to a discrepancy of 1.7 mm. Overall, 95 % of the keypoints exhibit a registration error of under 0.4 mm.

#### Impact of the scanning speed

**Fig. 8:**
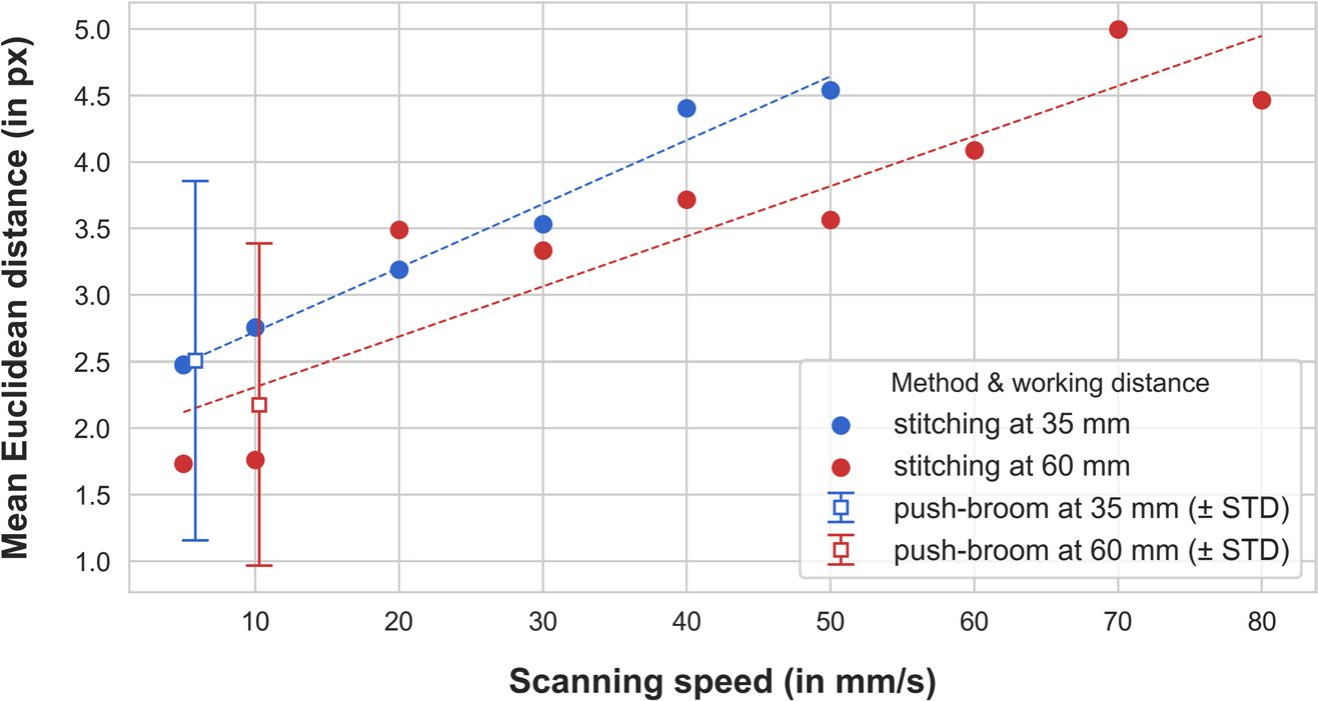
Distribution of the registration error with regard to the scanning speed. At a working distance of 35 mm, the stitching algorithm failed at speeds faster than 50 mm/s, so that no data are available for this distance at higher velocities. The blue and the red dashed lines correspond to the linear regression over the measured error values at both working distances. The squares depict the push-broom performance at both working distances, with error bars at one standard deviation. Samples: each point maps the registration error of one image, each square of five images, with each image averaged over 20 keypoint pairs (total: 340 keypoint pairs).

Figure 8 shows how robot-driven scan speed affects HSI panorama alignment with the RGB frame, revealing a linear correlation between speed and registration error. At a distance of 35 mm, the mean error increases from 2.5 px at 5 mm/s to 4.5 px at 50 mm/s. As for 60 mm, the mean errors range from 1.7 px at 5 mm/s to 6.0 px at 100 mm/s. Errors are generally higher at shorter distances, especially as speed increases. As for the push-broom, its performance roughly lines up at the lower range of the regression lines, with estimated speeds of 6 mm/s and 10 mm/s at working distances of 35 mm and 60 mm, respectively.

### Computational speed

Median processing times per frame and corresponding thoughput capacities of the assessed modules were 43 ms / 23 fps (local keypoints picking), 28 ms / 35 fps (global registration), 19 ms / 53 fps (line stitching), and 9 ms / 106 fps (visualization). Occasional spikes in the first three modules caused brief drops, with 6 fps as the rare worst-case minimum, but these delays were quickly compensated. The main runtime drivers were ORB keypoint extraction (80 % of picking time) and global feature matching (54 % of registration time). Over the minute-long sequences, processing times increased during the first 25 s but stabilized thereafter. This trend correlated with the sizes of the keypoint database and the panorama held in memory, both of which similarly plateaued thanks to regulated database pruning and automatic truncation of the panorama beyond the monitor view. Since both the database and the panorama account for most of the memory usage, their stabilization shows that overall memory consumption remains stable over time.

## Discussion

The qualitative examination acknowledged well-formed, expressive panoramas with a high level of detail, regardless of the scanning path or the working distance. Although presented in gray scales, the panoramas suggested that good readability and interpretation of hyperspectral data would be possible in a clinical context. Overall, the quantitative results confirmed these positive visual impressions and attested accurate panoramic reconstruction, with misregistration values comparable to those of push-broom scanning. In 95 % of the cases, neither robot-guided nor freehand scans exceeded a registration error of 5 px, amounting to less than half a millimeter on a real object at a realistic working distance of 50 mm. Furthermore, the pipeline showcased real-time capability, delivering smooth and fluid HSI visualization at a frame rate of 10 fps. Occasional minor lagging did not detract from the real-time video-like experience. This performance remained consistent and stable over time.

Yet, the qualitative examination of the panoramas revealed a few artifacts. A zigzag pattern was observed in some horizontal straight lines of the USAF target, appearing slightly jagged. This effect likely results from minor camera movements at frequencies exceeding the RGB sensor’s frame rate, which the homography transformation cannot fully correct. A ghosting effect was also noted, where light shadows appeared offset from areas of high contrast. Since this effect was also present in push-broom images, it seems to be an inherent characteristic of hyperspectral acquisition rather than a result of the stitching algorithm. Lastly, intensity discrepancies were observed in freehand panoramas, where overlapping sections showed visible seams due to varying pixel intensities. This artifact stems from vignetting, a reduction in brightness toward the image periphery, and is further influenced by variations in working distance, particularly when moving closer to the object. Such variations are common in laparoscopic settings, where precise distance control is not always feasible. To address this, physiological parameter calculations rely on spectral ratios rather than absolute intensities, making them largely robust to these fluctuations. By keeping the spectrometer slit fixed at the image center—where illumination is highest—our approach achieves a higher spectral signal-to-noise ratio than conventional push-broom systems. Furthermore, since the entire spectral information of each pixel is captured simultaneously, the scanning process does not affect spectral shape, even if spatial registration is imperfect. This spectral integrity is a notable advantage over other scanning approaches. Future work will focus on systematically quantifying the influence of intensity fluctuations on spectral integrity and parameter estimation.

On a spatial level, minor misregistration sometimes occurs, primarily due to the accumulation of several known systematic errors with similar effects on reconstruction, making it difficult to disentangle their individual contributions. One source of error is the spectrograph position, as the automated centering of the spectrograph’s entrance slit via the integrated motor varies slightly with each GUI restart. This requires periodic adjustment of line coordinates for stitching, leading to minor offsets. Another factor is barrel distortion, where residual radial distortion in RGB frames, despite calibration efforts, can affect homography accuracy. Although the central positioning of the spectrograph minimizes the impact, minor uncorrected distortions in HSI lines may still accumulate toward the panorama’s periphery. Additionally, a delay between RGB and HSI data appears to be present, as the raw images sent from both sensors to the Ethernet connection seem to be separated by a minimal time lag. While this delay was empirically estimated and compensated for in the algorithm, its accuracy and consistency over time remain uncertain.

Despite the overall accuracy of the reconstruction, a deeper analysis of the freehand scan uncovered an unexpected small set of error outliers, all located in the left half of the green square in Fig. 5b. This area, stitched early in the sequence around 20 seconds prior, exhibits notable drift from cumulative minor registration errors. In contrast, the right half, representing the latest scanned data, shows minimal error. This discrepancy is also responsible for the offset noted in the top red circle. In principle, the global frame-to-panorama registration significantly reduces drift, but the keypoints database’s forget-function, designed to limit its size by deleting older keypoints, can constrain the effectiveness of global registration. However, such misalignment is unlikely in live scenarios, as the function truncating areas outside the FOV would remove older regions potentially causing registration errors.

We also acknowledge that increasing scanning speed presents challenges with the current hardware setup. Higher speeds likely amplify systematic errors, leading to a decline in registration accuracy. The greater the displacement of a physical landmark between consecutive RGB frames, the higher the systematic error. In our experiments, accuracy decreased linearly, as only constant translations with the robotic arm were assessed. However, at higher velocities, misregistration is expected to escalate more rapidly, particularly with additional transformations such as rotations. Further, black gaps appear between HSI lines in empty canvas regions when the spacing between consecutive lines exceeds the width of a single line. The adaptive line width effectively eliminates this artifact, but its primary role is to improve visual continuity and readability—the underlying accuracy loss due to higher speeds remains unchanged. Lastly, at very high scanning speeds, the stitching algorithm can fail, producing distorted panoramas due to incorrect homography matrix estimates. Contributing factors include insufficient overlap for reliable keypoint matching, feature redundancy, uneven feature distribution, and vignetting effects that hinder keypoint detection. While plausibility checks filter out many incorrect estimations, some erroneous transformations persist. However, these shortcomings stem from the bandwidth constraints of the Ethernet connection, which limits data transmission between the laparoscope camera and the image processing computer. In the future, embedding image processing directly into the camera will eliminate this bottleneck, significantly increasing frame rate and system performance.

Despite demonstrating robustness in our experimental setting, the proposed method has currently three key limitations restricting its clinical applicability. First, the geometric transformation model assumes a planar scene. This assumption holds to some extent, as the RGB camera’s frame rate, combined with slow scanning motion, results in minimal shifts between frames. However, the model cannot capture complex spatial transformations in scenes with significant depth variation. In surgical scenarios, parallax effects and distortions caused by depth changes can degrade accuracy^22^. Second, our targets contained evenly distributed, detail-rich features that facilitated effective traditional keypoint detection. However, traditional keypoint detectors perform poorly in textureless areas, leading to keypoint clustering in high-contrast regions^22^. This limitation becomes clinically relevant when scanning structurally uniform tissues or organs, such as the liver. Finally, although the stitching pipeline is relatively resilient to motion artifacts compared to push-broom imaging, occlusions and deformations during stitching might still introduce distortions. In our future research, we will investigate the extent to which parallax and textureless regions affect registration, and assess the impact of dynamic factors—such as patient breathing and pulsating blood vessels—on overall accuracy.

Nevertheless, we believe that the main strength of our approach lies in the overall RGB/HSI co-registration framework, which provides a solid real-time running foundation for integrating more advanced techniques. Its modular structure allows for replacing individual components to address current limitations. In particular, deep learning models hold great promise for enhancing accuracy and reliability—whether by replacing traditional feature extractors and matchers, moving beyond the single homography principle to handle depth-varying images with parallax, or incorporating non-rigid transformations to account for local deformations in image registration. Yet, deep learning algorithms are computationally expensive, posing challenges for real-time processing. A promising approach would involve an adaptive stitching algorithm that dynamically switches between traditional methods and neural networks based on processing conditions. Such a hybrid pipeline would combine the speed of our current algorithm with the power of neural networks. Our future work will explore these directions to enhance pipeline robustness and adaptability, advancing it toward clinical applicability.

## Conclusion

We introduced a stitching algorithm for the HSI MIS system as an alternative to the push-broom motor-based hyperspectral acquisition method. Our real-time pipeline processes incoming RGB video and HSI data, enabling users to visualize hyperspectral information as a dynamically expanding overlay by manually scanning the scene with the laparoscope. Evaluations demonstrate that our method achieves accuracy comparable to the push-broom approach while offering several advantages. Operators can rapidly capture hyperspectral data from a smaller region of interest without waiting for a full-field scan, increasing system flexibility. Additionally, the algorithm continuously updates previously scanned areas, enabling real-time refreshment of hyperspectral information by revisiting regions. By dynamically adjusting the central line area of the panorama, our approach also helps mitigate image distortions and motion artifacts caused by patient movement. Overall, this method transforms hyperspectral visualization from a static to a dynamic process, enhancing usability. Its compatibility with emerging applications such as robotic surgery further underscores its potential to replace the current push-broom acquisition technique.

## Supplementary Information

Supplementary Information.

## Data Availability

The data is available from the corresponding author upon reasonable request.

## References

[1] Barberio, M. et al. Intraoperative guidance using hyperspectral imaging: A review for surgeons. *Diagnostics* **11**. 10.3390/diagnostics11112066 (2021). 10.3390/diagnostics11112066 PMC8624094 34829413

[2] Köhler, H. et al. Laparoscopic system for simultaneous high-resolution video and rapid hyperspectral imaging in the visible and near-infrared spectral range. *J. Biomed. Opt.* **25**, 1–13. 10.1117/1.JBO.25.8.086004 (2020). 10.1117/1.JBO.25.8.086004 PMC7453262 32860357

[3] Holmer, A., Marotz, J., Wahl, P., Dau, M. & Kämmerer, P. W. Hyperspectral imaging in perfusion and wound diagnostics–methods and algorithms for the determination of tissue parameters. *Biomed. Eng. Biomed. Tech.* **63**, 547–556. 10.1515/bmt-2017-0155 (2018). 10.1515/bmt-2017-0155 30028724

[4] Köhler, H. et al. Comparison of image registration methods for combining laparoscopic video and spectral image data. *Sci. Rep.* **12** (2022). 10.1038/s41598-022-20816-1. 10.1038/s41598-022-20816-1 PMC9525266 36180520

[5] Yoon, J. et al. A clinically translatable hyperspectral endoscopy (HySE) system for imaging the gastrointestinal tract. *Nat. Commun*. **10** (2019). 10.1038/s41467-019-09484-4. 10.1038/s41467-019-09484-4 PMC6478902 31015458

[6] Yoon, J. et al. First experience in clinical application of hyperspectral endoscopy for evaluation of colonic polyps. *J. Biophoton.* **14** (2021). 10.1002/jbio.202100078. 10.1002/jbio.202100078 34047490

[7] Szeliski, R. Image alignment and stitching: A tutorial. *Found. Trends. Comput. Graph. Vis.* **2**, 1–104. 10.1561/0600000009 (2006).

[8] Arth, C., Klopschitz, M., Reitmayr, G. & Schmalstieg, D. Real-time self-localization from panoramic images on mobile devices. In *2011 10th IEEE International Symposium on Mixed and Augmented Reality*. 37–46 (2011). 10.1109/ISMAR.2011.6092368.

[9] Schneider, R. J. et al. Real-time image-based rigid registration of three-dimensional ultrasound. *Med. Image Anal.* **16**, 402–414. 10.1016/j.media.2011.10.004 (2012). 22154960 10.1016/j.media.2011.10.004 PMC3267836

[10] Lin, Y. & Medioni, G. Map-enhanced UAV image sequence registration and synchronization of multiple image sequences. In *2007 IEEE Conference on Computer Vision and Pattern Recognition*. 1–7 (2007). 10.1109/CVPR.2007.383428.

[11] Bergen, T., Nowack, S., Münzenmayer, C. & Wittenberg, T. A hybrid tracking approach for endoscopic real-time panorama imaging. In *17th Annual Conference of the International Society for Computer Aided Surgery, Heidelberg, Germany*. *Int. J. CARS* **8**(Suppl. 01), 352–354 (Springer, 2013). 10.1007/s11548-013-0881-z.

[12] Bergen, T. *Real-Time Endoscopic Image Stitching for Cystoscopy* (Cuvillier Verlag Göttingen, 2017).

[13] The Qt Company. Qt Documentation (2021). https://doc.qt.io/qt-5. Accessed 24 Mar 2025.

[14] Bradski, G. The OpenCV library - Feature matching. *Dr. Dobb’s J. Softw. Tools* **25**, 120–125 (2000).

[15] Zuiderveld, K. J. Contrast limited adaptive histogram equalization. In *Graphics Gems*. 474–485 (1994). 10.1016/B978-0-12-336156-1.50061-6.

[16] Rublee, E., Rabaud, V., Konolige, K. & Bradski, G. ORB: An efficient alternative to SIFT or SURF. In *2011 International Conference on Computer Vision*. 2564–2571 (IEEE, 2011). 10.1109/ICCV.2011.6126544.

[17] Hartley, R. & Zisserman, A. *Multiple View Geometry in Computer Vision*. 2 Ed. (Cambridge University Press, 2004). 10.1017/CBO9780511811685.

[18] Fischler, M. A. & Bolles, R. C. Random sample consensus: A paradigm for model fitting with applications to image analysis and automated cartography. *Commun. ACM* **24**, 381–395. 10.1145/358669.358692 (1981).

[19] Baum, I. USAF-1951 test target (2013). https://commons.wikimedia.org/wiki/File:USAF-1951.svg. Accessed 24 Mar 2025.

[20] Moulla, Y. et al. Hybridösophagektomie mit intraoperativem Hyperspektral-Imaging: Videobeitrag. *Der Chirurg* **91**, 1–12. 10.1007/s00104-020-01139-1 (2020). 10.1007/s00104-020-01139-1 32067066

[21] Eberhardt, W. Kuka AG, Germany (2022). www.kuka.com/en-de/products/robot-systems/industrial-robots/lbr-iiwa. Accessed 24 Mar 2025.

[22] Szeliski, R. *Computer Vision – Algorithms and Applications. Texts in Computer Science*. 2 Ed. (Springer Cham, 2022). 10.1007/978-3-030-34372-9.

